# Expression and functional analysis of citrus carotene hydroxylases: unravelling the xanthophyll biosynthesis in citrus fruits

**DOI:** 10.1186/s12870-016-0840-2

**Published:** 2016-06-29

**Authors:** Gang Ma, Lancui Zhang, Witchulada Yungyuen, Issei Tsukamoto, Natsumi Iijima, Michiru Oikawa, Kazuki Yamawaki, Masaki Yahata, Masaya Kato

**Affiliations:** Department of Biological and Environmental Sciences, Faculty of Agriculture, Shizuoka University, 836 Ohya, Shizuoka, Suruga 422-8529 Japan; The United Graduate school of Agricultural Science, Gifu University (Shizuoka University), Yanagido, Gifu, 501-1193 Japan

**Keywords:** β-Cryptoxanthin, Flavedo, Juice sacs, Lutein, Satsuma mandarin

## Abstract

**Background:**

Xanthophylls are oxygenated carotenoids and fulfill critical roles in plant growth and development. In plants, two different types of carotene hydroxylases, non-heme di-iron and heme-containing cytochrome P450, were reported to be involved in the biosynthesis of xanthophyll. Citrus fruits accumulate a high amount of xanthophylls, especially β,β-xanthophylls. To date, however, the roles of carotene hydroxylases in regulating xanthophyll content and composition have not been elucidated.

**Results:**

In the present study, the roles of four carotene hydroxylase genes (*CitHYb*, *CitCYP97A*, *CitCYP97B*, and *CitCYP97C*) in the biosynthesis of xanthophyll in citrus fruits were investigated. Phylogenetic analysis showed that the four citrus carotene hydroxylases presented in four distinct clusters which have been identified in higher plants. CitHYb was a non-heme di-iron carotene hydroxylase, while CitCYP97A, CitCYP97B, and CitCYP97C were heme-containing cytochrome P450-type carotene hydroxylases. Gene expression results showed that the expression of *CitHYb* increased in the flavedo and juice sacs during the ripening process, which was well consistent with the accumulation of β,β-xanthophyll in citrus fruits. The expression of *CitCYP97A* and *CitCYP97C* increased with a peak in November, which might lead to an increase of lutein in the juice sacs during the ripening process. The expression level of *CitCYP97B* was much lower than that of *CitHYb*, *CitCYP97A*, and *CitCYP97C* in the juice sacs during the ripening process. Functional analysis showed that the CitHYb was able to catalyze the hydroxylation of the β-rings of β-carotene and α-carotene in *Escherichia coli* BL21 (DE3) cells. Meanwhile, when CitHYb was co-expressed with CitCYP97C, α-carotene was hydroxylated on the β-ring and ε-ring sequentially to produce lutein.

**Conclusions:**

*CitHYb* was a key gene for β,β-xanthophyll biosynthesis in citrus fruits. *CitCYP97C* functioned as an ε-ring hydroxylase to produce lutein using zeinoxanthin as a substrate. The results will contribute to elucidating xanthophyll biosynthesis in citrus fruits, and provide new strategies to improve the nutritional and commercial qualities of citrus fruits.

**Electronic supplementary material:**

The online version of this article (doi:10.1186/s12870-016-0840-2) contains supplementary material, which is available to authorized users.

## Background

Carotenoids are a diverse group of pigments widely distributed in nature that provide distinct colors to fruits and flowers, and fulfill critical roles in plant growth and development [1–4]. In nature, more than 700 carotenoids have been identified and divided into two groups: carotenes and xanthophylls. Carotenes are liner or cyclic hydrocarbons, and xanthophylls are oxygenated derivatives of carotenes, such as lutein, β-cryptoxanthin, zeaxanthin, and astaxanthin. In higher plants, xanthophylls play an important role in the photosynthesis and photoprotection. They are structural elements of the photosynthetic apparatus, and the xanthophyll cycle (lutein, zeaxanthin, and antheraxanthin) protects plants from the damage of high light irradiation by dissipating excess light energy [5–8]. In addition, xanthophylls can be oxidatively cleaved in a site-specific manner, producing different apocarotenoids with important metabolic functions, such as plant hormones, pigments, as well as aroma and scent compounds [9–13]. Xanthophylls are not only important to the plants themselves, but also beneficial to human health. Epidemiological studies suggested that xanthophylls, such as lutein, β-cryptoxanthin, and astaxanthin, were effective to prevent eye diseases, certain cancers and inflammation because of their high antioxidant activity [14–21].

**Fig. 1:**
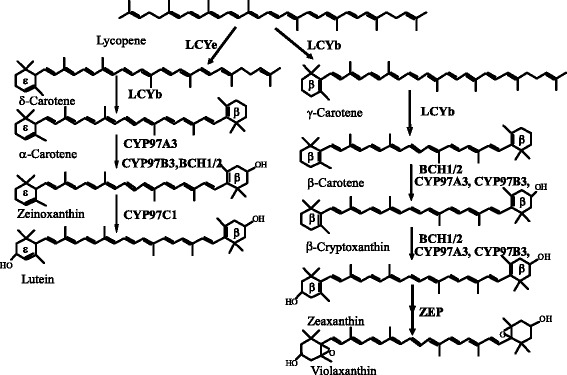
Xanthophyll biosynthetic pathway in Arabidopsis. LCYb, lycopene β-cyclase; LCYe, lycopene ε-cyclase; BCH1/2, β-ring hydroxylase1/2; CYP97A3, heme-containing cytochromes P450 monooxygenases A3; CYP97B3 heme-containing cytochromes P450 monooxygenases B3; CYP97C1, heme-containing cytochrome P450 monooxygenases C1; ZEP, zeaxanthin epoxidase

In plants, two different types of carotene hydroxylases, non-heme di-iron carotene hydroxylase and heme-containing cytochrome P450-type carotene hydroxylase, are involved in the biosynthesis of xanthophyll. Non-heme di-iron carotene hydroxylase (also called BCH, HYD, or HYb) efficiently catalyzes the hydroxylation of the β-rings of β-carotene (Fig. 1). In some plants species, it has been reported that two members of non-heme di-iron carotene hydroxylase existed, which had similar functions but tissue-specific expression patterns [22–25]. In Arabidopsis, a double-null mutation of BCH1 and BCH2 led to a significant decrease of β,β-xanthophylls [24, 26]. Recently, three heme-containing cytochrome P450-type carotene hydroxylases (CYP97A3, CYP97B3, and CYP97C1) have been identified in Arabidopsis. As shown in Fig. 1, CYP97C1 encoded by the *LUT1* locus in Arabidopsis is responsible for the ε-ring hydroxylation [27, 28]. CYP97C1 is a key enzyme for the biosynthesis of lutein, and its activity can not be replaced by other carotene hydroxylases. In tomato, up-regulation of *CYP97C11* led to an increase in the content of lutein in leaves. In contrast, when *CYP97C11* was down-regulated, lutein was almost absent (0.8 %) in tomato leaves [29]. CYP97A3 encoded by *LUT5* locus exhibits a major activity towards the β-ring of α-carotene, and a minor activity towards the β-rings of β-carotene in Arabidopsis [28, 30] (Fig. 1). Quinlan et al. [31] reported that OsCYP97A4 interacted with OsCYP97C2 in maize protoplasts, and the synergistic interaction between OsCYP97A4 and OsCYP97C2 drove the formation of lutein. Unlike CYP97A and CYP97C, the roles of CYP97B in the xanthophyll biosynthesis are still poorly studied. It has been suggested that CYP97B3 might be able to hydroxylate the β-rings of β-carotene and α-carotene in Arabidopsis [32, 33]. However, in the quadruple mutant (*bch1*, *bch2*, *cyp97c1*, and *cyp97a3*) that contained only CYP97B3, xanthophylls did not accumulated, indicating that CYP97B might not be an important enzyme for carotene hydroxylation [8, 24].

Citrus fruits accumulate a high amount of xanthophylls, especially β,β-xanthophylls, which account for up to 90 % of total carotenoids [34, 35]. In the previous studies, carotenoid metabolism has been extensively investigated in the fruits of different citrus varieties [11, 34–38]. Meanwhile, some key carotenoid metabolic genes have been isolated and their functions were deeply investigated in citrus fruits [9, 11, 12, 37, 39]. To date, however, the roles of carotene hydroxylases in regulating carotenoid content and composition are still unclear in citrus. In the present study, the changes in the expression of four carotene hydroxylase genes (*CitHYb*, *CitCYP97A*, *CitCYP97B*, and *CitCYP97C*) were investigated in the flavedo and juice sacs during the ripening process. In addition, to elucidate their roles in xanthophyll biosynthesis, functional analyses of the four carotene hydroxylase genes were conducted in *Escherichia coli* cells accumulating with different carotenoids. The results present in this study will contribute to further elucidating the mechanism of carotenoid accumulation in citrus fruits, and provide new insights into enhancing the nutritional and commercial qualities of citrus fruits.

## Results

### Isolation and characterization of carotene hydroxylase genes in citrus fruits

**Fig. 2:**
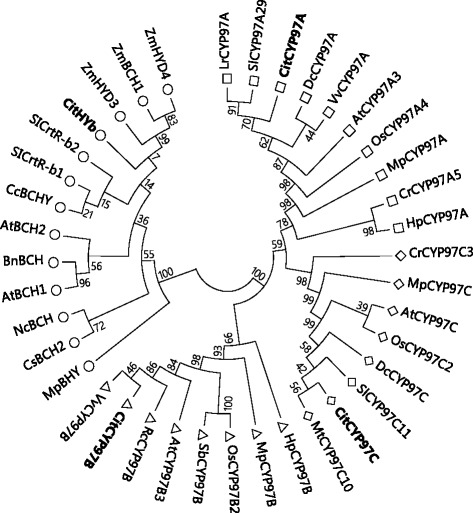
Phylogenetic analysis of carotene hydroxylases. The Neighbor-joining phylogenetic tree was constructed based on the alignment of the deduced amino acid sequences of carotene hydroxylases using MEGA6 software

In order to identify the carotene hydroxylase genes in citrus, we performed blast searches in the Citrus clementina v.10 and Citrus sinesis v 1.1 genome databases (http://www.phytozome.net/) using the sequences of Arabidopsis *BCH1*, *BCH2*, *CYP97A3*, *CYP97B3*, and *CYP97C1* as queries, respectively. Four carotene hydroxylase genes (*HYb*, *CYP97A*, *CYP97B*, and *CYP97C*) were identified in citrus genome database. In our previous study, *CitHYb* was isolated from Satsuma mandarin (Accession number: AB114653), while the information on *CYP97A*, *CYP97B*, and *CYP97C* in citrus fruits was completely unknown. In the present study, the full-length cDNAs of *CYP97A*, *CYP97B*, and *CYP97C* were isolated from Satsuma mandarin by RT-PCR using the primers designed within 5′ and 3′ UTRs according to the sequences obtained from the citrus genome database. The sequences of *CYP97A*, *CYP97B*, and *CYP97C* were named as *CitCYP97A*, *CitCYP97B*, and *CitCYP97C*, and submitted to the NCBI database (Accession numbers: *CitCYP97A*, LC143646; *CitCYP97B*, LC143647; *CitCYP97C*, LC143648). Phylogenetic analysis showed that the four citrus carotene hydroxylases presented in four distinct clusters which have been identified in higher plants (Fig. 2). CitHYb was a non-heme di-iron carotene hydroxylase, and its nucleotide sequence contained 936 bp, encoding a putative protein of 311 amino acids with a predicted molecular of 34.7 kDa. CitCYP97A, CitCYP97B, and CitCYP97C were heme-containing cytochrome P450-type carotene hydroxylases. The nucleotide sequence of *CitCYP97A* contained 1839 bp, and encoded a putative protein of 612 amino acids with a predicted molecular of 68.4 kDa. The nucleotide sequence of *CitCYP97B* contained 1749 bp, and encoded a putative protein of 582 amino acids with a predicted molecular of 65.2 kDa. The nucleotide sequence of *CitCYP97C* contained 1641 bp, and encoded a putative protein of 546 amino acids with a predicted molecular of 61.6 kDa. A chloroplastic transit peptide with different lengths was predicted in the N-terminal region of the proteins of CitHYb (62 amino acids), CitCYP97A (37 amino acids), CitCYP97B (50 amino acids), and CitCYP97C (18 amino acids).

### Changes in carotenoid contents and expression of carotene hydroxylase genes in the flavedo during the ripening process

**Fig. 3:**
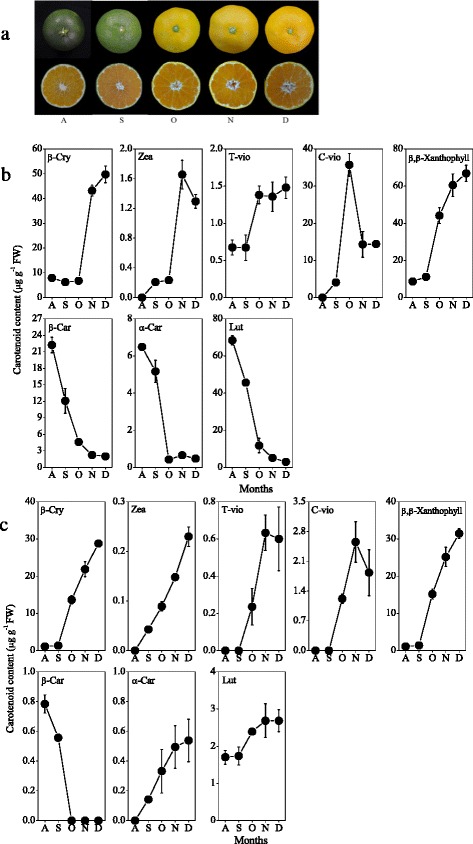
Carotenoid accumulation in the citrus fruits during the ripening process. **a** Changes in the colors of citrus fruits during the ripening process. **b** Changes in the carotenoid content in the flavedo during the ripening process. **c** Changes in the carotenoid content in the juice sacs during the ripening process. The results shown are the mean ± SE for triplicate samples. β-Car, β-carotene; β-Cry, β-cryptoxanthin; Zea, zeaxanthin; T-vio, all-*trans*-violaxanthin; C-vio, 9-*cis*-violaxanthin; α-Car, α-carotene; Lut, lutein; β,β-Xanthophyll, sum of β-Cry, Zea, T-vio and C-vio. A, August; S, September; O, October; N, November; D, December

**Fig. 4:**
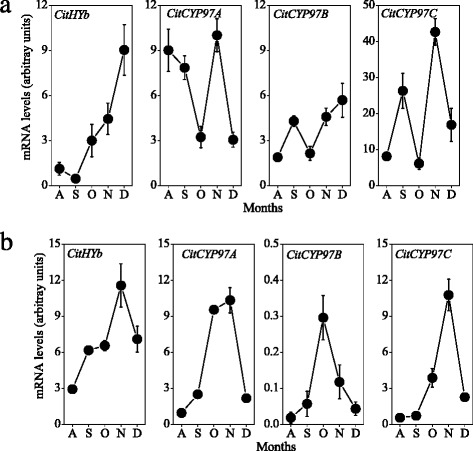
Changes in the expression of carotene hydroxylase genes in the flavedo (**a**) and juice sacs (**b**) during the ripening process. The results shown are the mean ± SE for triplicate samples. The mRNA levels were analyzed by TaqMan real-time quantitative RT-PCR. Real-time RT-PCR amplification of 18S rRNA was used to normalize the expression of the genes under identical conditions. A, August; S, September; O, October; N, November; D, December

In the present study, carotenoids were extracted from citrus fruits during the ripening process, and the changes in carotenoid content and composition were analyzed by HPLC. In the flavedo, the contents of β-carotene, α-carotene and lutein decreased rapidly from August, and then kept at a low level during the ripening process (Fig. 3a). The content of β-cryptoxanthin, the major carotenoid in Satsuma mandarin, increased significantly during the ripening process, and reached 49.7 μg g^−1^ in December. In addition, the contents of zeaxanthin, all-*trans*-violaxanthin and *cis*-violaxanthin also gradually increased, and as a result β,β-xanthophyll (sum of β-cryptoxanthin, zeaxanthin, all-*trans*-violaxanthin, and *cis*-violaxanthin) accumulated massively during the ripening process (Fig. 3b). Gene expression results showed that the expression of *CitHYb* increased gradually from September, which was consistent with the accumulation of β,β-xanthophyll during the ripening process (Fig. 4a). The expression of *CitCYP97A* decreased rapidly to a low level in October, and then increased with a peak in November during the ripening process. The expression of *CitCYP97C* increased with two peaks in September and November, respectively. Similarly to *CitCYP97C*, the expression of *CitCYP97B* increased gradually with a peak in September.

### Changes in carotenoid contents and expression of carotene hydroxylase genes in the juice sacs during the ripening process

In the juice sacs, the content of β-carotene decreased rapidly to an extremely low level in October, while the contents of β-cryptoxanthin and zeaxanthin increased significantly during the ripening process (Fig. 3c). The contents of α-carotene and lutein increased gradually in the juice sacs during the ripening process. Gene expression results showed that the expression of *CitHYb* increased in the juice sacs during the ripening process, which was in parallel with the accumulation of β,β-xanthophyll (Fig. 4b). The expression of *CitCYP97A*, *CitCYP97B*, and *CitCYP97C* increased with a peak in October and November, respectively. In addition, the expression level of *CitCYP97B* was much lower than that of *CitCYP97A* and *CitCYP97C* in the juice sacs during the ripening process (Fig. 4b).

### Functional analysis of carotene hydroxylase genes in *E. coli* cells

**Fig. 5:**
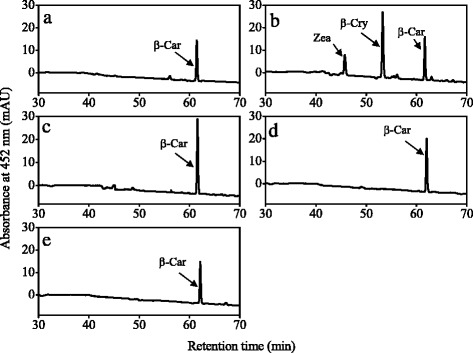
HPLC analysis of carotenoids in β-carotene-accumulating *E. coli* BL21 (DE3) cells transformed with pRSF-2 Ek/LIC-CitHYb (**b**), pRSF-2 Ek/LIC-CitCYP97A (**c**), pRSF-2 Ek/LIC-CitCYP97B (**d**), and pRSF-2 Ek/LIC-CitCYP97C (**e**). Carotenoids extracted from the suspension cultures of β-carotene-accumulating *E. coli* BL21 (DE3) cells with pRSF-2 (empty vector) were used as control (**a**). β-Car, β-carotene; β-Cry, β-cryptoxanthin; Zea, zeaxanthin

**Fig. 6:**
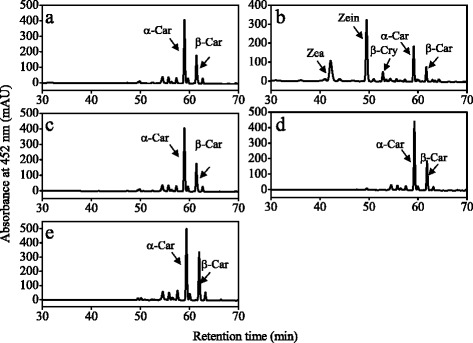
HPLC analysis of carotenoids in α-carotene and β-carotene-accumulating *E. coli* BL21 (DE3) cells transformed with pRSF-2 Ek/LIC-CitHYb (**b**), pRSF-2 Ek/LIC-CitCYP97A (**c**), pRSF-2 Ek/LIC-CitCYP97B (**d**), and pRSF-2 Ek/LIC-CitCYP97C (**e**). Carotenoids extracted from the suspension cultures of α-carotene and β-carotene-accumulating *E. coli* BL21 (DE3) cells with pRSF-2 (empty vector) were used as control (**a**). β-Car, β-carotene; β-Cry, β-cryptoxanthin; Zea, zeaxanthin; Zein, zeinoxanthin

In the present study, the cDNAs of *CitHYb*, *CitCYP97A*, *CitCYP97B*, and *CitCYP97C* without transcript peptides were cloned into pRSF-2 Ek/LIC vector, respectively. The four recombinant plasmids were transformed into β-carotene-accumulating *E. coli* BL21 (DE3) cells, as well as α-carotene- and β-carotene-accumulating *E. coli* BL21 (DE3) cells, respectively. Carotenoids were extracted from bacteria and analyzed by HPLC. When CitHYb was expressed in the β-carotene-accumulating *E. coli* BL21 (DE3) cells, the peaks of β-cryptoxanthin and zeaxanthin were observed (Fig. 5b). When CitHYb was expressed in the α-carotene- and β-carotene-accumulating *E. coli* BL21 (DE3) cells, a monohydroxylated intermediate, zeinoxanthin, was also detected, except for β-cryptoxanthin and zeaxanthin (Fig. 6b). In contrast to CitHYb, CitCYP97A, CitCYP97B, and CitCYP97C did not exhibit any carotene hydroxylation activity in the β-carotene-accumulating *E. coli* BL21 (DE3) cells, or α-carotene- and β-carotene-accumulating *E. coli* BL21 (DE3) cells (Figs. 5c, d, e and 6c, d, e).

**Fig. 7:**
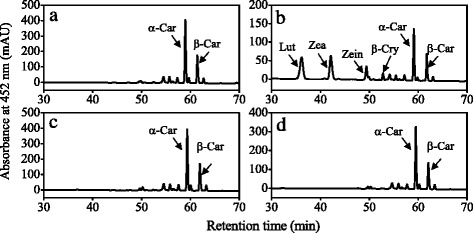
HPLC analysis of carotenoids in α-carotene and β-carotene-accumulating *E. coli* BL21 (DE3) cells co-transformed with pCDF-2 Ek/LIC-CitCYP97C and pRSF-2 Ek/LIC-CitHYb (**b**), pCDF-2 Ek/LIC-CitCYP97C and pRSF-2 Ek/LIC-CitCYP97A (**c**), pCDF-2 Ek/LIC-CitCYP97C and pRSF-2 Ek/LIC-CitCYP97B (**d**). Carotenoids extracted from the suspension cultures of α-carotene and β-carotene-accumulating *E. coli* BL21 (DE3) cells with pRSF-2 (empty vector) were used as control (**a**). β-Car, β-carotene; β-Cry, β-cryptoxanthin; Zea, zeaxanthin; Zein, zeinoxanthin; Lut, lutein

To further investigate the functions of carotene hydroxylase genes of citrus, we co-transformed *CitCYP97C* with *CitHYb*, *CitCYP97A*, and *CitCYP97B*, respectively. The recombinant plasmids were expressed in the α-carotene- and β-carotene-accumulating *E. coli* BL21 (DE3) cells. As shown in Fig. 7b, when CitHYb and CitCYP97C were co-expressed, the monohydroxylated zeinoxanthin, which was produced by CitHYb, was further converted to lutein by CitCYP97C. However, when CitCYP97A and CitCYP97C were co-expressed or CitCYP97B and CitCYP97C were co-expressed, no hydroxylated carotene was detected in the α-carotene- and β-carotene-accumulating *E. coli* BL21 (DE3) cells (Fig. 7c, d).

## Discussion

### Isolation and characterization of carotene hydroxylase genes in citrus fruits

Carotene hydroxylases were the key enzymes responsible for xanthophyll biosynthesis in plants. Two different types of carotene hydroxylases, non-heme di-iron carotene hydroxylase and heme-containing cytochrome P450-type carotene hydroxylase, have been identified in higher plants. In the present study, the roles of four carotene hydroxylase genes (*CitHYb*, *CitCYP97A*, *CitCYP97B*, and *CitCYP97C*) in regulating xanthophylls biosynthesis were investigated in citrus fruits. As shown in Fig. 2, the four carotene hydroxylase genes were clustered in distinct groups. CitHYb was a non-heme di-iron carotene hydroxylase, while CitCYP97A, CitCYP97B, and CitCYP97C were heme-containing cytochrome P450-type carotene hydroxylases. It has been reported that two or more *HYb* genes existed in some plant species, such as pepper, tomato, and Arabidopsis [23, 25, 40–42]. However, only one *HYb* was identified and isolated from citrus fruits, and it showed approximately 70 % identities to Arabidopsis *BCH1* and *BCH2* at the amino acid level. In the previous studies, the changes in the expression of *HYb* were extensively investigated in citrus fruits during the ripening process and under different environmental conditions [11, 34, 35, 43, 44]. In the juice sacs, different expression levels of *CitHYb* led to distinct carotenoid compositions between Satsuma mandarin and Valencia orange [34]. In contrast to *CitHYb*, the roles of heme-containing cytochrome P450-type carotene hydroxylases in regulating carotenoid accumulation in citrus fruits are completely unknown. In this study, it was the first time to isolate *CitCYP97A*, *CitCYP97B*, and *CitCYP97C* from the citrus fruits. Phylogenetic analysis suggested that CitCYP97A was more closely related to CitCYP97C than to CitCYP97B. CitCYP97B contained three insertions in the amino acid sequence compared with CitCYP97A and CitCYP97C, and shared around 42 % amino acid identity with CitCYP97A and CitCYP97C (Additional file 1: Figure S1). It has been reported that CYP97B3 in Arabidopsis was an uncharacterized cytochrome P450 monooxygenase, and the three amino acid insertions differentiated CYP97B from CYP97A and CYP97C [33, 45]. In addition, a transit peptide was predicted in the N-terminal reign of proteins encoded by *CitHYb*, *CitCYP97A*, *CitCYP97B*, and *CitCYP97C*, which suggested that the four carotene hydroxylases of citrus were able to import into plastids. As most carotenoids are synthesized and stored in plastids, the location of *CitHYb*, *CitCYP97A*, *CitCYP97B*, and *CitCYP97C* within plastid allows them to catalyze the reaction of carotene hydroxylation.

### Changes in carotenoid contents and expression of carotene hydroxylase genes in citrus fruits during the ripening process

In the previous studies, non-heme di-iron carotene hydroxylases were isolated from different plant species, and their roles in the carotenoid biosynthesis have been characterized [23–25, 46]. In Arabidopsis, BCH1 and BCH2 primarily catalyzed the hydroxylation of the β-rings of β-carotene [22, 47]. Du et al. [48] reported that DSM2 gene (BCH) controlled the biosyntheses of zeaxanthin and ABA, and conferred drought and oxidative stress resistance in rice. In citrus fruits, massive accumulation of xanthophylls, especially β,β-xanthophylls, occurred during the ripening process. In the present study, the results showed that the expression of *CitHYb* increased gradually in the flavedo and juice sacs during the ripening process (Fig. 4). The increase in the expression of *CitHYb* was well in agreement with the accumulation of β,β-xanthophyll in the flavedo and juice sacs (Fig. 3). This result was consistent with the findings of Pons et al. [49], in which inhibiting the expression of *Csβ-CHX* (*HYb*) by RNA interference led to a significant increase of β-carotene (up to 36-fold) and a decrease of β,β-xanthophylls in sweet orange. Thus, it was suggested that *CitHYb* was a key gene for β,β-xanthophyll biosynthesis in citrus fruits.

In contrast to non-heme di-iron carotene hydroxylase, heme-containing cytochrome P450-type carotene hydroxylases preferentially hydroxylated the β- and α-rings of α-carotene, yielding lutein in Arabidopsis [30]. Quinlan et al. [31] found that a synergistic interaction existed between rice OsCYP97A4 and OsCYP97C2, which was required to drive the biosynthesis of lutein. In the present study, gene expression results showed that the expression of *CitCYP97A* and *CitCYP97C* increased with a peak in November in juice sacs, which might lead to an increase of lutein during the ripening process (Figs. 3c and 4b). In the flavedo, the expression of *CitCYP97A* decreased rapidly from August, which was in parallel with the reduction of lutein in the green stage. In the orange stage (from October), the expression of *CitCYP97A* and *CitCYP97C* increased with a peak in November, while the content of lutein decreased to a low level in the flavedo (Figs. 3b and 4a). In citrus fruits, a change from β,ε-carotenoid accumulation (α-carotene and lutein) to β,β-carotenoid accumulation (β-carotene, β-cryptoxanthin, zeaxanthin, all-*trans*-violaxanthin, and *cis*-violaxanthin) was observed in the flavedo during the ripening process, accompanying the disappearance of *CitLCYe* transcripts and the increase in *CitLCYb* transcripts. We previously reported that the expression of *CitLCYe* decreased rapidly to a low level in the orange stage in the flavedo of Satsuma mandarin [34, 39]. Thus, it was suggested that the level of lutein in the orange stage was mainly controlled by *CitLCYe* instead of *CitCYP97A* and *CitCYP97C* in the flavedo.

CYP97B is another member of CYP97 family with carotene hydroxylation activity. CYP97B3 of Arabidopsis exhibited a potential hydroxylation activity towards β-carotene and α-carotene [32, 33]. However, it remains controversial whether CYP97B is involved in the xanthophyll biosynthesis, because xanthophylls did not accumulate in the Arabidopsis quadruple mutant (*bch1*, *bch2*, *cyp97c1*, and *cyp97a3*) with only CYP97B3 [8, 24]. In the present study, the results showed that the expression level of *CitCYP97B* in the juice sacs was much lower than that of *CitHYb*, *CitCYP97A*, and *CitCYP97C*, indicating that *CitCYP97B* might not be a key gene for carotene hydroxylation in the juice sacs of citrus fruits (Fig. 4b).

### Functional analysis of carotene hydroxylase genes in *E. coli* cells

In citrus, it is difficult to investigate the gene functions in the transgenic fruits because of its long juvenile phase that delays fruiting for 5–15 years [50]. As an alternative, *E. coli* cells that accumulate different carotenoids have been identified to be an efficient platform for investigating the functions of citrus carotenoid metabolic genes [11, 39, 43]. In the present study, we used the β-carotene-accumulating *E. coli* BL21 (DE3) cells and α-carotene- and β-carotene-accumulating *E. coli* BL21 (DE3) cells to investigate the functions of the four carotene hydroxylase genes of citrus. The results showed that CitHYb catalyzed the hydroxylation of the β-rings of β-carotene and α-carotene in *E. coli* BL21 (DE3) cells. When CitHYb was expressed in β-carotene-accumulating *E. coli* BL21 (DE3) cells, β-carotene was converted to β-cryptoxanthin and zeaxanthin, which supported the finding that *CitHYb* was a key gene for β,β-xanthophyll accumulation in citrus fruits (Fig. 5b). In Arabidopsis, it was reported that CYP97A3 and CYP97C1 were involved in lutein biosynthesis through hydroxylation of β- and ε-rings of α-carotene, respectively [24, 31]. However, in the absence of CYP97A the biosynthesis of lutein was not completely blocked. In Arabidopsis and rice, mutant of CYP97A only reduced around 20 % lutein compared with WT, which indicated that other carotene hydroxylases must also be able to catalyze hydroxylation of α-carotene on the β-ring [30, 51]. In the present study, we found that CitHYb participated in the biosynthesis of lutein. It converted α-carotene to zeinoxanthin, and the zeinoxanthin was further hydroxylated by CitCYP97C to produce lutein. Interestingly, CitCYP97C exhibited ε-ring hydroxylation activity only when it was co-expressed with CitHYb. Moreover, zeinoxanthin was the substrate for CitCYP97C instead of α-carotene, because α-cryptoxanthin, a monohydroxylated α-carotene on the ε-ring, was not detected in *E. coli* cells that transformed with *CitCYP97C* (Fig. 6e). These results suggested that α-carotene was hydroxylated on the β-ring and ε-ring sequentially to produce lutein in citrus fruits. A similar result was also reported in Arabidopsis, tomato and liverwort [29, 30, 52].

In contrast to the CitHYb, the carotene hydroxylation activities of CitCYP97A and CitCYP97B were not detected in *E. coli* BL21 (DE3) cells. In higher plants, only rice OsCYP97A4 was reported to exhibit β-ring hydroxylation activity in *E. coli* cells [31]. Amino acid sequence analysis showed that CitCYP97A shared 70 % similarity with OsCYP97A4, and the conserved oxygen-binding and heme-binding domains detected in CitCYP97A were identical to those of OsCYP97A4 (Additional file 2: Figure S2). In addition, we tested the activity of the full-length CitCYP97A, and optimized the culturing temperature and IPTG concentration as suggested in the study of Quinlan [45] (data not shown). Unfortunately, we could not detect any hydroxylation activity of CitCYP97A in *E. coli* cells. Similarly, attempts to assay the carotene hydroxylation activity of Arabidopsis CYP97A3 and liverwort MpCYP97A in *E. coli* cells were also failed [30, 52]. Whereas, mutant studies showed that Arabidopsis CYP97A3 (*LUT5* locus) exhibited a major hydroxylation activity towards the β-ring of α-carotene, and a minor activity towards the β-rings of β-carotene [30]. In orange carrot, a deficient *CYP97A3* allele caused the accumulation of α-carotene and a high α-/β-carotene ratio [53]. The research of Pons et al. [49] suggested that a second carotene hydroxylase might be present in sweet orange, because inhibiting the expression of *Csβ-CHX* (*HYb*) by RNA interference only caused a slight decrease in xanthophylls. In our present study, the gene expression results showed that *CitCYP97A* expressed in the flavedo and juice sacs during the ripening process. Meanwhile, the changes in *CitCYP97A* expression were consistent with the accumulation of lutein in the juice sacs (during the ripening stage) and flavedo (in the green stage), which indicating that *CitCYP97A* might be involved in the biosynthesis of lutein in citrus fruits. However, the mechanism that CitCYP97A regulates xanthophyll biosynthesis in citrus fruits seems to be more complicated, and some co-factors that are absent in *E. coli* cells might be needed for CitCYP97A to exert its activities. In the future research, further identification of the co-factors for heme-containing cytochrome P450-type carotene hydroxylases will contribute to elucidating the role of *CitCYP97A* in carotenoid accumulation.

## Conclusion

In the present study, the roles of the four carotene hydroxylase genes (*CitHYb*, *CitCYP97A*, *CitCYP97B*, and *CitCYP97C*) in regulating xanthophyll biosynthesis were investigated in citrus fruits. The results showed that *CitHYb* was a key gene for β,β-xanthophyll biosynthesis in citrus fruits. Functional analysis showed that the CitHYb was able to hydroxylate the β-rings of β-carotene and α-carotene in *E. coli* BL21 (DE3) cells. Meanwhile, when CitHYb was co-expressed with CitCYP97C, α-carotene was hydroxylated on the β-ring and ε-ring sequentially to produce lutein. In addition, we detected the expression of *CitCYP97A* in citrus fruits during the ripening process, and the changes in its expression were consistent with the accumulation of lutein in the juice sacs (during the ripening stage) and flavedo (in the green stage), which indicating that *CitCYP97A* might be involved in the biosynthesis of lutein in citrus fruits. The results presented in this study will contribute to further elucidating the mechanism of carotenoid biosynthesis in citrus fruits, and provide new strategies to improve carotenoid composition of citrus fruits.

## Methods

### Plant material

Satsuma mandarin (*Citrus unshiu* Marc.) cultivated at the Fujieda Farm of Shizuoka University (Shizuoka, Japan) were used as materials. Fruit samples were collected periodically from August to December. The flavedo and juice sacs were separated from sampled fruits, immediately frozen in liquid nitrogen, and kept at −80 °C until used.

### Extraction and determination of carotenoids

The identification and quantification of carotenoids were conducted according to the methods described by Ma et al. [11]. Pigments were extracted from the samples using a hexane:acetone:ethanol (2:1:1 [v/v]) solution containing 0.1 % (w/v) 2,6-di-*tert*-butyl-4-methylphenol and 10 % (w/v) magnesium carbonate basic. After the organic solvents had been completely evaporated, the extracts containing carotenoids esterified to fatty acids were saponified with 20 % (w/v) methanolic KOH. Water-soluble extracts were then removed by adding NaCl-saturated water. The pigments repartitioned into the diethylether phase were recovered and evaporated to dryness. Subsequently, the residue was redissolved in 5 mL of a TBME: methanol (1:1 [v/v]) solution. An aliquot (20 μL) was separated by a reverse-phase HPLC system (Jasco, Tokyo, Japan) fitted with a YMC Carotenoid S-5 column of 250- × 4.6-mm-i.d. (Waters, Milford, MA) at a flow rate of 1 mL min^−1^. The eluent was monitored by a photodiode array detector (MD-2015, Jasco). The carotenoid concentration was estimated by the standard curves and expressed as milligrams per gram fresh weight [34]. Carotenoid quantification was performed in three replicates.

### Isolation and sequence analysis of carotene hydroxylase genes

Total RNA was extracted from the flavedo of Satsuma mandarin fruits according to the method described by Ikoma et al. [54]. First-strand cDNA was synthesized from 2 μg of total RNA using TaqMan Reverse Transcription Reagents (Applied Biosystems). The full-length cDNAs of *CitCYP97A*, *CitCYP97B*, and *CitCYP97C* were amplified by RT-PCR using the primers designed within 5′ and 3′ UTRs according to the sequences obtained from the citrus genome database (Additional file 3: Table S1). The amplified cDNAs were cloned into the TOPO TA vector and sequenced using a BigDye Terminator v3.1 Cycle Sequencing Kit (Applied Biosystems, Foster City, CA, USA) with an ABI PRISM 3100 Genetic Analyzer (Applied Biosystems).

The alignment of *CitHYb*, *CitCYP97A*, *CitCYP97B*, and *CitCYP97C* was created using CLUSTAL W (http://www.clustal.org). The Neighbor-joining phylogenetic tree was constructed based on the alignment of the deduced amino acid sequences of carotene hydroxylases using MEGA6 software [55]. Accession numbers are: Arabidopsis AtBCH1, AY113923; Arabidopsis AtBCH2, AY117225; Brassica napus BnBCH, EF026098; Coffea canephora CcBCHY, DQ157165; Citrus CitHYb1, AB114653; Crocus sativus CsBCH2, AY579207; Lycopersicon esculentum SICrtR-b1, Y14809; Lycopersicon esculentum SICrtR-b2, Y14810; Marchantia polymorpha MpBHY, AB981062; Narcissus tazetta var. chinensis NcBCH, JN625263; Zea mays ZmBCH1, GQ131287; Zea mays ZmHYD3, AY844958; Zea mays ZmHYD4, AY844956; Arabidopsis AtCYP97A3, NM_102914; Chlamydomonas reinhardtii CrCYP97A5, EF587911; Citrus CitCYP97A, LC143646; Daucus carota DcCYP97A3, JQ655297; Haematococcus pluvialis HpCYP97A, JX308236; Lycopersicon esculentum SlCYP97A29, EU849605; Lycium ruthenicum LrCYP97A, KF957714; Marchantia polymorpha MpCYP97A, AB981063; Oryza sativa OsCYP97A4, AK068163; Vitis vinifera VvCYP97A, XP_002279984; Arabidopsis AtCYP97C1, AY424805; Citrus CitCYP97C, LC143648; Chlamydomonas reinhardtii CrCYP97C3, EF587910; Daucus carota DcCYP97C, ABB52076; Lycopersicon esculentum SlCYP97C11, EU849604; Medicago truncatula MtCYP97C10, ABC59096; Marchantia polymorpha MpCYP97C, AB981065; Oryza sativa OsCYP97C2, AK065689; Arabidopsis AtCYP97B3, NM_117600; Citrus CitCYP97B, LC143647; Haematococcus pluvialis HpCYP97B, JX272918; Marchantia polymorpha MpCYP97B, AB981064; Oryza sativa OsCYP97B2, XM_015771315; Ricinus communis RcCYP97B, XP_002520583; Sorghum bicolor SbCYP97B, XP_002451628; Vitis vinifera VvCYP97B, XP_002266883. Predictions of transit peptides of *CitHYb*, *CitCYP97A*, *CitCYP97B*, and *CitCYP97C* were carried out using TargetP.

### Total RNA extraction and real-time quantitative RT-PCR

Total RNA was extracted from the flavedo and juice sacs of Satsuma mandarin at different stages according to the method described by Ikoma et al. [54]. The total RNA was cleaned up using the RNeasy Mini Kit (Qiagen) with on-column DNase digestion. The reverse transcription (RT) reaction was performed with 2 μg of purified RNA and a random hexamer at 37 °C for 60 min using TaqMan Reverse Transcription Reagents (Applied Biosystems).

TaqMan MGB probes and sets of primers for *CitHYb*, *CitCYP97A*, *CitCYP97B*, and *CitCYP97C* were designed with the Primer Express software (Additional file 4: Table S2). For the endogenous control, the TaqMan Ribosomal RNA Control Reagents VIC Probe (Applied Biosystems) was used. TaqMan real-time PCR was carried out with the TaqMan Universal PCR Master Mix (Applied Biosystems) using ABI PRISM 7300 (Applied Biosystems) according to the manufacturer’s instructions. Each reaction contained 900 nM of the primers, 250 nM of the TaqMan MGB Probe, and template cDNA. The thermal cycling conditions were 95 °C for 10 min followed by 40 cycles of 95 °C for 15 s and 60 °C for 60 s. The levels of gene expression were analyzed with ABI PRISM 7300 Sequence Detection System Software (Applied Biosystems) and normalized with the results of 18S ribosomal RNA. Real-time quantitative RT-PCR was performed in three replicates for each sample.

### Functional analysis of the carotene hydroxylases in *E. coli* cells

The cDNAs of *CitHYb*, *CitCYP97A*, *CitCYP97B*, and *CitCYP97C* without transit peptide were cloned into the pRSF-2 Ek/LIC vector or pCDF-2 Ek/LIC vector, respectively. In the previous study, two recombinant plasmids pET-*CitLCYb1* and pET-*CitLCYb1* + *CitLCYe* were constructed, and transformed into *E. coli* BL21 (DE3) cells harboring a lycopene biosynthetic palsmid pACCRT-*EIB*, respectively [39, 56]. The co-transformation of pACCRT-*EIB* with pET-*CitLCYb1* or pET-*CitLCYb1* + *CitLCYe* led to β-carotene accumulation or α-carotene and β-carotene-accumulation in the *E. coli* BL21 (DE3) cells. In the present study, the recombinant plasmids pRSF-2-*CitHYb*, pRSF-2-*CitCYP97A*, pRSF-2-*CitCYP97B*, and pRSF-2-*CitCYP97C* were transformed into the β-carotene-accumulating, as well as α-carotene and β-carotene-accumulating *E. coli* BL21 (DE3) cells. To investigate the interactions among the carotene hydroxylases of citrus, we co-expressed pCDF-2-*CitCYP97C* with pRSF-2-*CitHYb*, pRSF-2-*CitCYP97A*, and pRSF-2-*CitCYP97B* in the α-carotene and β-carotene-accumulating *E. coli* BL21 (DE3) cells, respectively. After induction with 0.05 M isopropyl β-D-thiogalactoside (IPTG) for 2 d at 27 °C, carotenoids were extracted from *E. coli* cells. Cultures of *E. coli* cells were centrifuged at 5,000 g for 10 min and the bacterial pellet was washed twice with Tris–HCl (pH 8.0). The pellet was dried using vacuum freeze drying and stored at −20 °C until the HPLC analysis. The freeze-ground material was extracted with a mixture of chloroform and methanol (2:1 [v/v]) until all the color was removed from the *E. coli* cells. The carotenoid extracts were reduced to dryness by rotary evaporation, and then dissolved in the methyl *tert*-butyl ether: methanol (1:1 [v/v]) solution containing 0.1 % butylated hydroxyl toluene. α-Carotene, β-carotene, β-cryptoxanthin, zeaxanthin, and lutein were identified by comparing their specific retention times and absorption spectra with the authentic standards (Kato et al. 2004). The identification of zeinoxanthin was conducted using the methods described by Meléndez-Martínez et al. [57]. For each carotene hydroxylase gene, three replicates were conducted using different colonies in the functional analysis in *E. coli* cells.

### Statistical analysis

All values are shown as the mean ± SE for three replicates. The data were analyzed, and Tukey’s HSD test (at *P* < 0.05) was used to compare the treatment means.

## Abbreviations

CYP, cytochrome P450; HYb, β-ring hydroxylase; LCYb, lycopene β-cyclase; LCYe, lycopene ε-cyclase; ZEP, zeaxanthin expoxidase.

## Additional files

Additional file 1: Figure S1. Three sequence insertions in CitCYP97B compared with that of CitCYP97A and CitCYP97C. (DOCX 38 kb) Additional file 2: Figure S2. Alignment of deduced amino acid sequences of CitCYP97A and OsCYP97A4. The alignment was created using CLUSTAL W (http://www.clustal.org). (DOCX 153 kb) Additional file 3: Table S1. Primer sequences used for isolating full-length cDNAs of *CitCYP97A*, *CitCYP97B*, *CitCYP97C*. (DOCX 12 kb) Additional file 4: Table S2. Primer sequences and TaqMan MGB Probes used for the quantitative RT-PCRs of carotene hydroxylase genes. (DOCX 38 kb)
