# Supplementary material for: Expression and functional analysis of citrus carotene hydroxylases: unravelling the xanthophyll biosynthesis in citrus fruits
Source: BMC Plant Biol. 2016 Jun 29;16:148. doi: 10.1186/s12870-016-0840-2 (PMC4928310; doi:10.1186/s12870-016-0840-2)
Supplement: Additional file 1: Figure S1. — Three sequence insertions in CitCYP97B compared with that of CitCYP97A and CitCYP97C. (DOCX 38 kb) [file 12870_2016_840_MOESM1_ESM.docx]

**Additional file 1: Figure S1.** Three sequence insertions in CitCYP97B compared with that of CitCYP97A and CitCYP97C.

|  | Insertion 1 | Insertion 2 | Insertion 3 |
| --- | --- | --- | --- |
| CitCYP97B | KFEKLLEG-EDSR | PGGYRGDKDGYPVP | LKPRKDVGIEGWSGFDPSRSPGAL |
| AtCYP97B | KSEKLIREKETSS | PGGHKGEKEGHKVP | LRTKESNGIEGWAGFDPSRSPGAL |
| CitCYP97A | KFDTA--------ASE | LGKY-------------PIKR | PLD------------------------------------GPN |
| AtCYP97A | KLDAA--------ALK | LGKY-------------PIKR | PLD------------------------------------GPN |
| CitCYP97C | RLQTD--------ALN | PGNY ------------KVNA | DLE------------------------------------GPM |
| AtCYP97C | KLQPY--------AED | PGNY ------------KVNT | DID-------------------------------------GAI |
