# Supplementary material for: Expression and functional analysis of citrus carotene hydroxylases: unravelling the xanthophyll biosynthesis in citrus fruits
Source: BMC Plant Biol. 2016 Jun 29;16:148. doi: 10.1186/s12870-016-0840-2 (PMC4928310; doi:10.1186/s12870-016-0840-2)
Supplement: Additional file 2: Figure S2. — Alignment of deduced amino acid sequences of CitCYP97A and OsCYP97A4. The alignment was created using CLUSTAL W (http://www.clustal.org). (DOCX 153 kb) [file 12870_2016_840_MOESM2_ESM.docx]

**Additional file 2:** **Figure S2.** Alignment of deduced amino acid sequences of CitCYP97A and OsCYP97A4. The alignment was created using CLUSTAL W (http://www.clustal.org).
