# Supplementary material for: Expression and functional analysis of citrus carotene hydroxylases: unravelling the xanthophyll biosynthesis in citrus fruits
Source: BMC Plant Biol. 2016 Jun 29;16:148. doi: 10.1186/s12870-016-0840-2 (PMC4928310; doi:10.1186/s12870-016-0840-2)
Supplement: Additional file 3: Table S1. — Primer sequences used for isolating full-length cDNAs of CitCYP97A, CitCYP97B, CitCYP97C. (DOCX 12 kb) [file 12870_2016_840_MOESM3_ESM.docx]

**Additional file 3: Table S1.** Primer sequences used for isolating full-length cDNAs of *CitCYP97A*, *CitCYP97B*, *CitCYP97C*.

| cDNA | Position | Sequence |
| --- | --- | --- |
| *CitCYP97A* | Sense primer | AGGAGGCTGATAAAATTCTTGCGTTTCATT |
|  | Antisense primer | TAGGCATTTAGCCTCTCTTAATGTAGCAAT |
| *CitCYP97B* | Sense primer | TCATTTGGCTGTGGTGTGGCTCACTG |
|  | Antisense primer | GTTGAAAACTATTACAGGTTCTCCTTACTG |
| *CitCYP97C* | Sense primer | GTGTTAAGCACCGGAGATGCTCTTACTGCA |
|  | Antisense primer | TGCGGAGCATAGCCGAATGAATGATCTCCA |
