# Supplementary material for: Expression and functional analysis of citrus carotene hydroxylases: unravelling the xanthophyll biosynthesis in citrus fruits
Source: BMC Plant Biol. 2016 Jun 29;16:148. doi: 10.1186/s12870-016-0840-2 (PMC4928310; doi:10.1186/s12870-016-0840-2)
Supplement: Additional file 4: Table S2. — Primer sequences and TaqMan MGB Probes used for the quantitative RT-PCRs of carotene hydroxylase genes. (DOCX 38 kb) [file 12870_2016_840_MOESM4_ESM.docx]

**Additional file 4: Table S2.** Primer sequences and TaqMan MGB Probes used for the quantitative RT-PCRs of carotene hydroxylase genes.

| cDNA | Position | Sequence |
| --- | --- | --- |
| *CitHYb* | Sense primer | GCGGCTCACCAGCTTCAC |
|  | Antisense primer | CCGAGAAAGAGCCCATATGG |
|  | TaqMan MGB Probe | ACTCGGATAAATTCC |
| *CitCYP97A* | Sense primer | TGGAGGGCCACGGAAAT |
|  | Antisense primer | CGCAACTGCTACTATGTTCTCAAAG |
|  | TaqMan MGB Probe | CGTAGGTGATATGTTTGCT |
| *CitCYP97B* | Sense primer | GAAACGACGGCTGCTGTTC |
|  | Antisense primer | TTGAGGGATTTTGAGCCAGAA |
|  | TaqMan MGB Probe | ACTTGGGCTGTTTTT |
| *CitCYP97C* | Sense primer | TCAATGCGTCTCTACCCACATC |
|  | Antisense primer | TTTCCAGGGAGCACATCATCT |
|  | TaqMan MGB Probe | TCCTGTCTTGATAAGAAG |
